# 1,1,1-Tris(di­methyl­amino)-2-[tris­(di­methyl­amino)­phospho­ranyl­idene]diphosphinium tetra­phenyl­borate tetra­hydro­furan monosolvate

**DOI:** 10.1107/S1600536814011258

**Published:** 2014-05-21

**Authors:** Bobby D. Ellis, Erica V. Morasset, Charles L. B. Macdonald

**Affiliations:** a Department of Chemistry and Biochemistry, University of Windsor, Windsor, Ontario, N9B 3P4, Canada

## Abstract

In the tetra­hydro­furan solvate of the title salt, C_12_H_36_N_6_P_3_^+^·C_24_H_20_B^−^·C_4_H_8_O, the cation features short P—P bond lengths [2.1111 (11) and 2.1364 (10) Å] and a distinctly bent P—P—P angle [104.67 (4)°] that confirm that the mol­ecule is not allene-like. In the crystal, the solvent mol­ecule is linked to the cation *via* a weak C—H⋯O hydrogen bond.

## Related literature   

For the preparation of [P(P[NMe_3_])_2_][BPh_4_], see: Schmidpeter & Lochschmidt (1986 ▶). For reviews of triphosphenium and related low-oxidation-state group 15 mol­ecules, see: Ellis & Macdonald (2007 ▶); Coffer & Dillon (2013 ▶). For the use of [P(P[NMe_3_])_2_]^+^ salts as a source of P^+^, see: Schmidpeter *et al.* (1983 ▶); Driess *et al.* (1999 ▶); Schmidpeter (1999 ▶). For the structure of the only related acyclic triphosphenium salt [P(PPh_3_)_2_][AlCl_4_], see: Ellis & Macdonald (2006 ▶). For a related structure, see: Appel *et al.* (1983 ▶). For a description of the Cambridge Structural Database (CSD), see: Allen (2002 ▶).
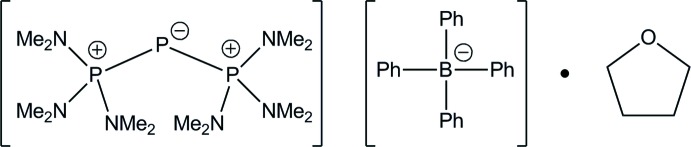


## Experimental   

### 

#### Crystal data  

C_12_H_36_N_6_P_3_^+^·C_24_H_20_B^−^·C_4_H_8_O
*M* *_r_* = 748.69
Monoclinic, 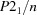

*a* = 13.0859 (17) Å
*b* = 11.8258 (16) Å
*c* = 27.504 (4) Å
β = 98.930 (2)°
*V* = 4204.7 (10) Å^3^
*Z* = 4
Mo *K*α radiation
μ = 0.18 mm^−1^
*T* = 183 K
0.50 × 0.30 × 0.30 mm

#### Data collection  

Bruker APEX CCD diffractometer
Absorption correction: multi-scan (*SADABS*; Bruker, 2000 ▶) *T*_min_ = 0.883, *T*_max_ = 0.948
47616 measured reflections
9592 independent reflections
5621 reflections with *I* > 2σ(*I*)
*R*_int_ = 0.090

#### Refinement  

*R*[*F* ^2^ > 2σ(*F* ^2^)] = 0.062
*wR*(*F* ^2^) = 0.154
*S* = 1.02
9592 reflections
472 parameters
H-atom parameters constrained
Δρ_max_ = 0.50 e Å^−3^
Δρ_min_ = −0.30 e Å^−3^

### 

Data collection: *SMART* (Bruker, 1997 ▶); cell refinement: *SAINT* (Bruker, 1997 ▶); data reduction: *SAINT*; program(s) used to solve structure: *SHELXS2012* (Sheldrick, 2008 ▶); program(s) used to refine structure: *SHELXL2012* (Sheldrick, 2008 ▶); molecular graphics: *SHELXTL* (Sheldrick, 2008 ▶); software used to prepare material for publication: *WinGX* (Farrugia, 2012 ▶) and *PLATON* (Spek, 2009 ▶).

## Supplementary Material

Crystal structure: contains datablock(s) I. DOI: 10.1107/S1600536814011258/lh5704sup1.cif

Structure factors: contains datablock(s) I. DOI: 10.1107/S1600536814011258/lh5704Isup2.hkl

Supporting information file. DOI: 10.1107/S1600536814011258/lh5704Isup3.cdx

CCDC reference: 1003430

Additional supporting information:  crystallographic information; 3D view; checkCIF report

## Figures and Tables

**Table 1:** Hydrogen-bond geometry (Å, °)

*D*—H⋯*A*	*D*—H	H⋯*A*	*D*⋯*A*	*D*—H⋯*A*
C11—H33⋯O1^i^	0.98	2.52	3.454 (4)	158

Symmetry code: (i) 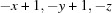.

**Fig. 1.**
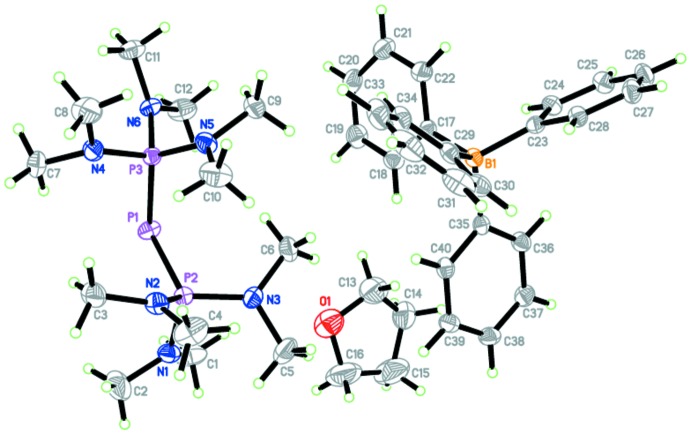
Displacement ellipsoid plot (40% probability surface) of the contents of the asymmetric unit of [P(P[NMe3])2][BPh4]·THF (1).

## supplementary crystallographic information

### 1. Comment

The triphosphenium salt [P(P[NMe_3_])_2_][BPh_4_], which was first reported by Schmidpeter & Lochschmidt (1986), has been used as a reagent and a source of "P^+^" for decades but no structure of any salt containing the cation has ever been reported. As part of our ongoing investigations of low valent group 15 compounds, we were able to obtain crystals of the tetrahydrofuran solvate of the salt (1) from the slow evaporation of a THF solution of the salt.

The molecular structure of (1) is shown in Fig. 1. The P–P distances in the cation in (1) of 2.1111 (11) Å and 2.1364 (10) Å are significantly shorter than typical P–P single bonds *ca* 2.24 (2) Å that have been reported in the Cambridge Structural Database (as determined from the 14 examples of diorganodiphosphines that are found in CSD Version 5.35) (Allen, 2002) but are consistent with those reported for salts of other triphosphenium cations (Ellis & Macdonald, 2007). More importantly, the P–P–P angle of 104.67 (4)° clearly indicates that the geometry about the dicoordinate phosphorus atom in (1) is best-described as being bent and thus resembles that of the only other structurally characterized triphosphenium (Ellis & Macdonald, 2006). The bent geometry in (1) is in stark contrast to the perfectly linear allene-like P–C–P arangement in the analogous carbodiphosphorane C(P[NMe_3_])_2_ (Appel *et al.*, 1983).

The metrical parameters of the tetraphenylborate anion and the THF solvent of crystallization are unexceptional and there are no unusually short cation-anion contacts. Details of a weak hydrogen bond between the cation and the THF oxygen atom (1) is listed in Table 1.

### 2. Experimental

The salt (1) was synthesized using the method described by Schmidpeter and Lochschmidt (1986). Suitable crystals were obtained by the slow evaporation of a tetrahydrofuran solution of the salt in a nitrogen-filled glove box. The crystal used for data collection was coated in mineral oil, mounted and placed in the cold stream on the diffractometer.

### 3. Refinement

All non-H atoms were refined anisotropically and H atoms were initially located in the difference Fourier maps. The H atoms were subsequently modeled as riding atoms with a C–H distance of 0.98 Å and *U(H)* of 1.5 times that of the carbon atom to which they are attached for each methyl hydrogen atom; each rigid methyl group was allowed to rotate in order to maximize the sum of electron density at the calculated H atom positions. The H atoms on the phenyl groups were modeled with a C–H distance of 0.95 Å and *U(H)* of 1.2 times that of the carbon atom to which they are attached and each methylene hydrogen atom was modeled with a C–H distance of 0.99 Å and *U(H)* of 1.2 times that of the carbon atom to which they are attached.

### Crystal data

**Table:**

C_12_H_36_N_6_P_3_^+^·C_24_H_20_B^−^·C_4_H_8_O	*F*(000) = 1616
*M**_r_* = 748.69	*D*_x_ = 1.183 Mg m^−^^3^
Monoclinic, *P*2_1_/*n*	Mo *K*α radiation, λ = 0.71073 Å
*a* = 13.0859 (17) Å	Cell parameters from 3924 reflections
*b* = 11.8258 (16) Å	θ = 2.4–22.8°
*c* = 27.504 (4) Å	µ = 0.18 mm^−^^1^
β = 98.930 (2)°	*T* = 183 K
*V* = 4204.7 (10) Å^3^	Block, colourless
*Z* = 4	0.50 × 0.30 × 0.30 mm

### Data collection

**Table:**

Bruker APEX CCD diffractometer	5621 reflections with *I* > 2σ(*I*)
φ and ω scans	*R*_int_ = 0.090
Absorption correction: multi-scan (*SADABS*; Bruker, 2000)	θ_max_ = 27.5°, θ_min_ = 2.3°
*T*_min_ = 0.883, *T*_max_ = 0.948	*h* = −16→16
47616 measured reflections	*k* = −15→15
9592 independent reflections	*l* = −34→35

### Refinement

**Table:**

Refinement on *F*^2^	0 restraints
Least-squares matrix: full	Hydrogen site location: inferred from neighbouring sites
*R*[*F*^2^ > 2σ(*F*^2^)] = 0.062	H-atom parameters constrained
*wR*(*F*^2^) = 0.154	*w* = 1/[σ^2^(*F*_o_^2^) + (0.0576*P*)^2^ + 2.6057*P*] where *P* = (*F*_o_^2^ + 2*F*_c_^2^)/3
*S* = 1.02	(Δ/σ)_max_ < 0.001
9592 reflections	Δρ_max_ = 0.50 e Å^−^^3^
472 parameters	Δρ_min_ = −0.30 e Å^−^^3^

### Special details

**Table:**

Geometry. All e.s.d.'s (except the e.s.d. in the dihedral angle between two l.s. planes) are estimated using the full covariance matrix. The cell e.s.d.'s are taken into account individually in the estimation of e.s.d.'s in distances, angles and torsion angles; correlations between e.s.d.'s in cell parameters are only used when they are defined by crystal symmetry. An approximate (isotropic) treatment of cell e.s.d.'s is used for estimating e.s.d.'s involving l.s. planes.

### Fractional atomic coordinates and isotropic or equivalent isotropic displacement parameters (Å^2^)

**Table:**

	*x*	*y*	*z*	*U*_iso_*/*U*_eq_	
C17	0.1618 (2)	0.7471 (2)	0.09042 (10)	0.0259 (6)	
C18	0.2657 (2)	0.7266 (2)	0.08915 (11)	0.0318 (7)	
H49	0.3101	0.7125	0.1193	0.038*	
C19	0.3078 (3)	0.7257 (3)	0.04550 (12)	0.0412 (8)	
H48	0.3793	0.7099	0.0464	0.049*	
C20	0.2467 (3)	0.7473 (3)	0.00135 (12)	0.0415 (8)	
H47	0.2753	0.7469	−0.0284	0.050*	
C21	0.1434 (3)	0.7696 (3)	0.00076 (12)	0.0423 (8)	
H46	0.1001	0.7848	−0.0296	0.051*	
C22	0.1024 (2)	0.7699 (3)	0.04441 (11)	0.0373 (8)	
H45	0.0309	0.7863	0.0431	0.045*	
C23	0.0343 (2)	0.8560 (2)	0.14270 (10)	0.0241 (6)	
C24	0.0602 (2)	0.9610 (2)	0.12407 (10)	0.0293 (7)	
H54	0.1192	0.9652	0.1079	0.035*	
C25	0.0040 (2)	1.0588 (2)	0.12824 (11)	0.0331 (7)	
H53	0.0257	1.1282	0.1158	0.040*	
C26	−0.0837 (2)	1.0558 (3)	0.15043 (11)	0.0357 (7)	
H52	−0.1230	1.1223	0.1533	0.043*	
C27	−0.1128 (2)	0.9543 (3)	0.16831 (11)	0.0343 (7)	
H51	−0.1732	0.9504	0.1835	0.041*	
C28	−0.0550 (2)	0.8575 (2)	0.16449 (10)	0.0274 (6)	
H50	−0.0773	0.7888	0.1773	0.033*	
C29	0.0420 (2)	0.6272 (2)	0.14288 (11)	0.0283 (7)	
C30	0.0050 (2)	0.5941 (3)	0.18592 (12)	0.0352 (7)	
H59	0.0209	0.6399	0.2145	0.042*	
C31	−0.0538 (2)	0.4971 (3)	0.18854 (14)	0.0455 (9)	
H58	−0.0788	0.4787	0.2182	0.055*	
C32	−0.0757 (3)	0.4277 (3)	0.14815 (16)	0.0531 (10)	
H57	−0.1157	0.3612	0.1498	0.064*	
C33	−0.0393 (2)	0.4551 (3)	0.10556 (15)	0.0483 (9)	
H56	−0.0530	0.4068	0.0777	0.058*	
C34	0.0177 (2)	0.5539 (2)	0.10316 (12)	0.0356 (7)	
H55	0.0410	0.5721	0.0731	0.043*	
C35	0.2023 (2)	0.7470 (2)	0.18904 (10)	0.0230 (6)	
C36	0.2409 (2)	0.8475 (2)	0.21153 (11)	0.0306 (7)	
H64	0.2098	0.9170	0.2000	0.037*	
C37	0.3232 (2)	0.8498 (3)	0.25017 (11)	0.0343 (7)	
H63	0.3472	0.9200	0.2643	0.041*	
C38	0.3701 (2)	0.7508 (3)	0.26809 (11)	0.0331 (7)	
H62	0.4261	0.7521	0.2946	0.040*	
C39	0.3344 (2)	0.6499 (3)	0.24694 (10)	0.0308 (7)	
H61	0.3661	0.5809	0.2587	0.037*	
C40	0.2523 (2)	0.6487 (2)	0.20859 (10)	0.0271 (6)	
H60	0.2289	0.5778	0.1949	0.033*	
B1	0.1095 (2)	0.7444 (3)	0.14112 (11)	0.0243 (7)	
P1	0.47131 (6)	0.27326 (7)	0.04361 (3)	0.0298 (2)	
P2	0.45906 (5)	0.24507 (6)	0.11828 (3)	0.02473 (17)	
P3	0.31635 (5)	0.26032 (6)	0.00575 (3)	0.02397 (17)	
N1	0.57948 (18)	0.2276 (2)	0.14651 (9)	0.0337 (6)	
N2	0.39109 (19)	0.1337 (2)	0.12853 (9)	0.0328 (6)	
N3	0.4218 (2)	0.3539 (2)	0.14747 (9)	0.0340 (6)	
N4	0.28536 (19)	0.1285 (2)	−0.00843 (9)	0.0346 (6)	
N5	0.21714 (18)	0.3064 (2)	0.03007 (9)	0.0315 (6)	
N6	0.32168 (18)	0.3316 (2)	−0.04498 (8)	0.0295 (6)	
C1	0.6525 (3)	0.3206 (3)	0.14466 (13)	0.0517 (10)	
H1	0.7029	0.3213	0.1750	0.078*	
H2	0.6149	0.3925	0.1414	0.078*	
H3	0.6888	0.3102	0.1164	0.078*	
C2	0.6274 (3)	0.1186 (3)	0.15207 (17)	0.0751 (14)	
H4	0.6570	0.1003	0.1224	0.113*	
H5	0.5756	0.0615	0.1569	0.113*	
H6	0.6824	0.1192	0.1807	0.113*	
C3	0.3938 (3)	0.0276 (3)	0.10199 (13)	0.0466 (9)	
H7	0.4200	−0.0325	0.1251	0.070*	
H8	0.4393	0.0356	0.0771	0.070*	
H9	0.3238	0.0084	0.0859	0.070*	
C4	0.3379 (3)	0.1247 (3)	0.17174 (12)	0.0533 (10)	
H10	0.2719	0.0853	0.1625	0.080*	
H11	0.3250	0.2007	0.1837	0.080*	
H12	0.3813	0.0824	0.1978	0.080*	
C5	0.4510 (3)	0.3687 (3)	0.20099 (11)	0.0502 (9)	
H13	0.4893	0.4397	0.2076	0.075*	
H14	0.4947	0.3053	0.2145	0.075*	
H15	0.3885	0.3710	0.2165	0.075*	
C6	0.3654 (3)	0.4497 (3)	0.12355 (13)	0.0499 (9)	
H16	0.2953	0.4509	0.1320	0.075*	
H17	0.3615	0.4428	0.0878	0.075*	
H18	0.4012	0.5199	0.1348	0.075*	
C7	0.3606 (3)	0.0557 (3)	−0.02720 (14)	0.0559 (10)	
H19	0.3548	−0.0214	−0.0148	0.084*	
H20	0.4305	0.0845	−0.0161	0.084*	
H21	0.3468	0.0552	−0.0633	0.084*	
C8	0.1787 (3)	0.0863 (3)	−0.02147 (14)	0.0571 (11)	
H22	0.1656	0.0667	−0.0565	0.086*	
H23	0.1299	0.1451	−0.0148	0.086*	
H24	0.1695	0.0190	−0.0018	0.086*	
C9	0.1719 (3)	0.4183 (3)	0.01855 (12)	0.0440 (9)	
H25	0.0973	0.4107	0.0077	0.066*	
H26	0.2039	0.4530	−0.0077	0.066*	
H27	0.1843	0.4661	0.0480	0.066*	
C10	0.1772 (2)	0.2494 (3)	0.07023 (13)	0.0492 (9)	
H28	0.1971	0.2919	0.1008	0.074*	
H29	0.2060	0.1728	0.0741	0.074*	
H30	0.1016	0.2450	0.0627	0.074*	
C11	0.2432 (2)	0.3102 (3)	−0.08840 (11)	0.0419 (8)	
H31	0.1839	0.3605	−0.0876	0.063*	
H32	0.2203	0.2313	−0.0881	0.063*	
H33	0.2731	0.3245	−0.1184	0.063*	
C12	0.3759 (3)	0.4398 (3)	−0.04721 (12)	0.0487 (9)	
H34	0.4152	0.4385	−0.0747	0.073*	
H35	0.4233	0.4517	−0.0164	0.073*	
H36	0.3253	0.5015	−0.0520	0.073*	
O1	0.5933 (2)	0.6186 (2)	0.17083 (10)	0.0677 (8)	
C13	0.5372 (3)	0.7229 (4)	0.16305 (16)	0.0684 (12)	
H43	0.4708	0.7173	0.1759	0.082*	
H44	0.5223	0.7406	0.1275	0.082*	
C14	0.6020 (4)	0.8106 (4)	0.18915 (16)	0.0716 (13)	
H41	0.5593	0.8685	0.2026	0.086*	
H42	0.6447	0.8480	0.1671	0.086*	
C15	0.6683 (4)	0.7486 (4)	0.22969 (18)	0.0956 (17)	
H39	0.7408	0.7751	0.2331	0.115*	
H40	0.6428	0.7602	0.2614	0.115*	
C16	0.6611 (5)	0.6294 (5)	0.21556 (16)	0.1004 (18)	
H37	0.7304	0.6003	0.2118	0.120*	
H38	0.6350	0.5846	0.2415	0.120*	

### Atomic displacement parameters (Å^2^)

**Table:**

	*U*^11^	*U*^22^	*U*^33^	*U*^12^	*U*^13^	*U*^23^
C17	0.0274 (14)	0.0208 (14)	0.0296 (15)	−0.0011 (12)	0.0042 (12)	−0.0038 (12)
C18	0.0311 (16)	0.0343 (17)	0.0304 (16)	0.0027 (13)	0.0057 (13)	0.0047 (13)
C19	0.0371 (18)	0.047 (2)	0.0429 (19)	0.0020 (15)	0.0165 (15)	−0.0004 (16)
C20	0.056 (2)	0.0383 (19)	0.0333 (17)	−0.0005 (17)	0.0174 (15)	−0.0030 (15)
C21	0.054 (2)	0.043 (2)	0.0294 (17)	0.0019 (17)	0.0034 (15)	−0.0026 (15)
C22	0.0306 (16)	0.045 (2)	0.0348 (17)	0.0036 (14)	0.0016 (13)	−0.0043 (15)
C23	0.0217 (15)	0.0258 (15)	0.0237 (15)	−0.0015 (11)	0.0000 (12)	−0.0032 (12)
C24	0.0249 (15)	0.0305 (16)	0.0327 (17)	−0.0010 (13)	0.0051 (13)	0.0019 (13)
C25	0.0353 (18)	0.0225 (16)	0.0408 (18)	−0.0025 (13)	0.0039 (15)	0.0022 (13)
C26	0.0319 (18)	0.0280 (17)	0.046 (2)	0.0079 (13)	0.0021 (15)	−0.0061 (14)
C27	0.0281 (16)	0.0364 (18)	0.0397 (18)	0.0014 (14)	0.0094 (14)	−0.0062 (15)
C28	0.0271 (15)	0.0246 (15)	0.0304 (16)	−0.0012 (12)	0.0046 (13)	−0.0013 (12)
C29	0.0183 (14)	0.0241 (15)	0.0418 (18)	0.0019 (12)	0.0022 (13)	0.0005 (13)
C30	0.0301 (17)	0.0299 (17)	0.0438 (19)	−0.0019 (13)	−0.0002 (14)	0.0079 (14)
C31	0.0295 (18)	0.042 (2)	0.065 (2)	−0.0014 (15)	0.0074 (17)	0.0213 (18)
C32	0.0296 (19)	0.0273 (18)	0.099 (3)	−0.0066 (15)	−0.002 (2)	0.012 (2)
C33	0.0339 (19)	0.0267 (17)	0.082 (3)	−0.0027 (15)	0.0006 (19)	−0.0161 (18)
C34	0.0261 (16)	0.0292 (17)	0.051 (2)	0.0002 (13)	0.0043 (15)	−0.0084 (15)
C35	0.0204 (13)	0.0243 (14)	0.0259 (14)	−0.0002 (12)	0.0086 (11)	0.0002 (12)
C36	0.0291 (16)	0.0256 (16)	0.0360 (17)	0.0002 (13)	0.0017 (14)	−0.0017 (13)
C37	0.0343 (18)	0.0307 (17)	0.0364 (18)	−0.0045 (14)	0.0008 (14)	−0.0089 (14)
C38	0.0279 (15)	0.0408 (18)	0.0287 (15)	−0.0006 (14)	−0.0013 (12)	0.0002 (14)
C39	0.0287 (16)	0.0332 (17)	0.0300 (17)	0.0033 (13)	0.0024 (13)	0.0044 (13)
C40	0.0261 (15)	0.0232 (15)	0.0330 (16)	−0.0018 (12)	0.0075 (13)	−0.0030 (12)
B1	0.0224 (15)	0.0217 (16)	0.0286 (16)	−0.0009 (13)	0.0030 (13)	−0.0015 (14)
P1	0.0225 (4)	0.0440 (5)	0.0234 (4)	−0.0045 (3)	0.0049 (3)	−0.0005 (3)
P2	0.0237 (4)	0.0262 (4)	0.0243 (4)	−0.0016 (3)	0.0038 (3)	0.0007 (3)
P3	0.0227 (4)	0.0240 (4)	0.0249 (4)	−0.0011 (3)	0.0027 (3)	−0.0015 (3)
N1	0.0287 (13)	0.0399 (16)	0.0311 (14)	0.0034 (12)	0.0003 (11)	0.0031 (12)
N2	0.0357 (15)	0.0282 (14)	0.0343 (14)	−0.0063 (11)	0.0045 (12)	0.0063 (11)
N3	0.0415 (15)	0.0341 (14)	0.0269 (14)	0.0022 (12)	0.0066 (12)	−0.0039 (11)
N4	0.0315 (14)	0.0265 (14)	0.0425 (16)	−0.0016 (11)	−0.0050 (12)	−0.0060 (11)
N5	0.0269 (13)	0.0354 (14)	0.0334 (14)	0.0040 (11)	0.0082 (11)	0.0041 (11)
N6	0.0308 (14)	0.0351 (14)	0.0216 (13)	−0.0021 (11)	0.0013 (10)	0.0006 (10)
C1	0.0332 (19)	0.077 (3)	0.042 (2)	−0.0181 (18)	−0.0039 (16)	0.0092 (19)
C2	0.051 (2)	0.061 (3)	0.101 (4)	0.021 (2)	−0.026 (2)	−0.025 (2)
C3	0.053 (2)	0.0266 (18)	0.056 (2)	−0.0037 (16)	−0.0066 (18)	0.0014 (16)
C4	0.060 (2)	0.060 (2)	0.042 (2)	−0.024 (2)	0.0142 (18)	0.0099 (18)
C5	0.066 (3)	0.054 (2)	0.0320 (19)	−0.0021 (19)	0.0127 (18)	−0.0094 (16)
C6	0.053 (2)	0.0355 (19)	0.057 (2)	0.0142 (17)	−0.0047 (18)	−0.0152 (17)
C7	0.068 (3)	0.038 (2)	0.056 (2)	0.0136 (18)	−0.007 (2)	−0.0202 (18)
C8	0.053 (2)	0.035 (2)	0.075 (3)	−0.0166 (17)	−0.019 (2)	−0.0012 (18)
C9	0.041 (2)	0.051 (2)	0.040 (2)	0.0186 (16)	0.0060 (16)	−0.0021 (16)
C10	0.0285 (17)	0.074 (3)	0.048 (2)	0.0008 (17)	0.0148 (15)	0.0164 (19)
C11	0.0394 (19)	0.058 (2)	0.0262 (17)	−0.0008 (17)	−0.0009 (14)	0.0013 (15)
C12	0.056 (2)	0.051 (2)	0.039 (2)	−0.0125 (18)	0.0026 (17)	0.0132 (17)
O1	0.083 (2)	0.0602 (19)	0.0594 (18)	−0.0155 (16)	0.0089 (16)	−0.0013 (14)
C13	0.047 (2)	0.094 (4)	0.065 (3)	0.005 (2)	0.007 (2)	−0.004 (3)
C14	0.097 (3)	0.054 (3)	0.063 (3)	0.013 (2)	0.011 (3)	−0.010 (2)
C15	0.115 (4)	0.096 (4)	0.064 (3)	−0.019 (3)	−0.026 (3)	−0.013 (3)
C16	0.154 (5)	0.090 (4)	0.047 (3)	0.002 (4)	−0.016 (3)	0.015 (3)

### Geometric parameters (Å, º)

**Table:**

C17—C18	1.388 (4)	N2—C4	1.471 (4)
C17—C22	1.405 (4)	N3—C6	1.453 (4)
C17—B1	1.646 (4)	N3—C5	1.472 (4)
C18—C19	1.397 (4)	N4—C7	1.461 (4)
C18—H49	0.9500	N4—C8	1.474 (4)
C19—C20	1.370 (4)	N5—C10	1.459 (4)
C19—H48	0.9500	N5—C9	1.464 (4)
C20—C21	1.375 (5)	N6—C12	1.470 (4)
C20—H47	0.9500	N6—C11	1.471 (4)
C21—C22	1.390 (4)	C1—H1	0.9800
C21—H46	0.9500	C1—H2	0.9800
C22—H45	0.9500	C1—H3	0.9800
C23—C28	1.394 (4)	C2—H4	0.9800
C23—C24	1.406 (4)	C2—H5	0.9800
C23—B1	1.650 (4)	C2—H6	0.9800
C24—C25	1.385 (4)	C3—H7	0.9800
C24—H54	0.9500	C3—H8	0.9800
C25—C26	1.381 (4)	C3—H9	0.9800
C25—H53	0.9500	C4—H10	0.9800
C26—C27	1.373 (4)	C4—H11	0.9800
C26—H52	0.9500	C4—H12	0.9800
C27—C28	1.385 (4)	C5—H13	0.9800
C27—H51	0.9500	C5—H14	0.9800
C28—H50	0.9500	C5—H15	0.9800
C29—C34	1.392 (4)	C6—H16	0.9800
C29—C30	1.402 (4)	C6—H17	0.9800
C29—B1	1.648 (4)	C6—H18	0.9800
C30—C31	1.390 (4)	C7—H19	0.9800
C30—H59	0.9500	C7—H20	0.9800
C31—C32	1.375 (5)	C7—H21	0.9800
C31—H58	0.9500	C8—H22	0.9800
C32—C33	1.370 (5)	C8—H23	0.9800
C32—H57	0.9500	C8—H24	0.9800
C33—C34	1.394 (4)	C9—H25	0.9800
C33—H56	0.9500	C9—H26	0.9800
C34—H55	0.9500	C9—H27	0.9800
C35—C36	1.398 (4)	C10—H28	0.9800
C35—C40	1.400 (4)	C10—H29	0.9800
C35—B1	1.648 (4)	C10—H30	0.9800
C36—C37	1.391 (4)	C11—H31	0.9800
C36—H64	0.9500	C11—H32	0.9800
C37—C38	1.376 (4)	C11—H33	0.9800
C37—H63	0.9500	C12—H34	0.9800
C38—C39	1.378 (4)	C12—H35	0.9800
C38—H62	0.9500	C12—H36	0.9800
C39—C40	1.383 (4)	O1—C16	1.406 (5)
C39—H61	0.9500	O1—C13	1.435 (5)
C40—H60	0.9500	C13—C14	1.456 (6)
P1—P2	2.1111 (11)	C13—H43	0.9900
P1—P3	2.1364 (10)	C13—H44	0.9900
P2—N3	1.631 (2)	C14—C15	1.495 (6)
P2—N2	1.638 (2)	C14—H41	0.9900
P2—N1	1.658 (2)	C14—H42	0.9900
P3—N6	1.641 (2)	C15—C16	1.461 (6)
P3—N4	1.642 (2)	C15—H39	0.9900
P3—N5	1.643 (2)	C15—H40	0.9900
N1—C2	1.432 (4)	C16—H37	0.9900
N1—C1	1.463 (4)	C16—H38	0.9900
N2—C3	1.454 (4)		
			
C18—C17—C22	114.7 (3)	C10—N5—C9	113.7 (2)
C18—C17—B1	123.8 (2)	C10—N5—P3	123.6 (2)
C22—C17—B1	121.5 (2)	C9—N5—P3	122.0 (2)
C17—C18—C19	122.8 (3)	C12—N6—C11	113.4 (2)
C17—C18—H49	118.6	C12—N6—P3	124.6 (2)
C19—C18—H49	118.6	C11—N6—P3	119.1 (2)
C20—C19—C18	120.4 (3)	N1—C1—H1	109.5
C20—C19—H48	119.8	N1—C1—H2	109.5
C18—C19—H48	119.8	H1—C1—H2	109.5
C19—C20—C21	118.9 (3)	N1—C1—H3	109.5
C19—C20—H47	120.5	H1—C1—H3	109.5
C21—C20—H47	120.5	H2—C1—H3	109.5
C20—C21—C22	120.1 (3)	N1—C2—H4	109.5
C20—C21—H46	119.9	N1—C2—H5	109.5
C22—C21—H46	119.9	H4—C2—H5	109.5
C21—C22—C17	122.9 (3)	N1—C2—H6	109.5
C21—C22—H45	118.5	H4—C2—H6	109.5
C17—C22—H45	118.5	H5—C2—H6	109.5
C28—C23—C24	114.3 (3)	N2—C3—H7	109.5
C28—C23—B1	124.4 (2)	N2—C3—H8	109.5
C24—C23—B1	121.2 (2)	H7—C3—H8	109.5
C25—C24—C23	123.1 (3)	N2—C3—H9	109.5
C25—C24—H54	118.4	H7—C3—H9	109.5
C23—C24—H54	118.4	H8—C3—H9	109.5
C26—C25—C24	120.2 (3)	N2—C4—H10	109.5
C26—C25—H53	119.9	N2—C4—H11	109.5
C24—C25—H53	119.9	H10—C4—H11	109.5
C27—C26—C25	118.5 (3)	N2—C4—H12	109.5
C27—C26—H52	120.8	H10—C4—H12	109.5
C25—C26—H52	120.8	H11—C4—H12	109.5
C26—C27—C28	120.7 (3)	N3—C5—H13	109.5
C26—C27—H51	119.7	N3—C5—H14	109.5
C28—C27—H51	119.7	H13—C5—H14	109.5
C27—C28—C23	123.2 (3)	N3—C5—H15	109.5
C27—C28—H50	118.4	H13—C5—H15	109.5
C23—C28—H50	118.4	H14—C5—H15	109.5
C34—C29—C30	115.0 (3)	N3—C6—H16	109.5
C34—C29—B1	124.0 (3)	N3—C6—H17	109.5
C30—C29—B1	121.1 (3)	H16—C6—H17	109.5
C31—C30—C29	122.7 (3)	N3—C6—H18	109.5
C31—C30—H59	118.6	H16—C6—H18	109.5
C29—C30—H59	118.6	H17—C6—H18	109.5
C32—C31—C30	119.9 (3)	N4—C7—H19	109.5
C32—C31—H58	120.0	N4—C7—H20	109.5
C30—C31—H58	120.0	H19—C7—H20	109.5
C33—C32—C31	119.6 (3)	N4—C7—H21	109.5
C33—C32—H57	120.2	H19—C7—H21	109.5
C31—C32—H57	120.2	H20—C7—H21	109.5
C32—C33—C34	119.9 (3)	N4—C8—H22	109.5
C32—C33—H56	120.1	N4—C8—H23	109.5
C34—C33—H56	120.1	H22—C8—H23	109.5
C29—C34—C33	122.9 (3)	N4—C8—H24	109.5
C29—C34—H55	118.5	H22—C8—H24	109.5
C33—C34—H55	118.5	H23—C8—H24	109.5
C36—C35—C40	114.9 (2)	N5—C9—H25	109.5
C36—C35—B1	122.7 (2)	N5—C9—H26	109.5
C40—C35—B1	122.3 (2)	H25—C9—H26	109.5
C37—C36—C35	122.6 (3)	N5—C9—H27	109.5
C37—C36—H64	118.7	H25—C9—H27	109.5
C35—C36—H64	118.7	H26—C9—H27	109.5
C38—C37—C36	120.5 (3)	N5—C10—H28	109.5
C38—C37—H63	119.8	N5—C10—H29	109.5
C36—C37—H63	119.8	H28—C10—H29	109.5
C37—C38—C39	118.8 (3)	N5—C10—H30	109.5
C37—C38—H62	120.6	H28—C10—H30	109.5
C39—C38—H62	120.6	H29—C10—H30	109.5
C38—C39—C40	120.2 (3)	N6—C11—H31	109.5
C38—C39—H61	119.9	N6—C11—H32	109.5
C40—C39—H61	119.9	H31—C11—H32	109.5
C39—C40—C35	123.1 (3)	N6—C11—H33	109.5
C39—C40—H60	118.5	H31—C11—H33	109.5
C35—C40—H60	118.5	H32—C11—H33	109.5
C17—B1—C29	109.7 (2)	N6—C12—H34	109.5
C17—B1—C35	109.0 (2)	N6—C12—H35	109.5
C29—B1—C35	108.9 (2)	H34—C12—H35	109.5
C17—B1—C23	109.6 (2)	N6—C12—H36	109.5
C29—B1—C23	110.3 (2)	H34—C12—H36	109.5
C35—B1—C23	109.4 (2)	H35—C12—H36	109.5
P2—P1—P3	104.67 (4)	C16—O1—C13	106.9 (3)
N3—P2—N2	109.77 (13)	O1—C13—C14	107.1 (3)
N3—P2—N1	101.69 (13)	O1—C13—H43	110.3
N2—P2—N1	108.73 (13)	C14—C13—H43	110.3
N3—P2—P1	115.34 (10)	O1—C13—H44	110.3
N2—P2—P1	114.81 (10)	C14—C13—H44	110.3
N1—P2—P1	105.37 (9)	H43—C13—H44	108.6
N6—P3—N4	109.09 (13)	C13—C14—C15	104.1 (4)
N6—P3—N5	108.44 (12)	C13—C14—H41	110.9
N4—P3—N5	103.30 (13)	C15—C14—H41	110.9
N6—P3—P1	102.43 (9)	C13—C14—H42	110.9
N4—P3—P1	111.38 (9)	C15—C14—H42	110.9
N5—P3—P1	121.87 (10)	H41—C14—H42	108.9
C2—N1—C1	113.7 (3)	C16—C15—C14	105.6 (4)
C2—N1—P2	122.1 (2)	C16—C15—H39	110.6
C1—N1—P2	118.0 (2)	C14—C15—H39	110.6
C3—N2—C4	113.3 (3)	C16—C15—H40	110.6
C3—N2—P2	123.4 (2)	C14—C15—H40	110.6
C4—N2—P2	122.3 (2)	H39—C15—H40	108.7
C6—N3—C5	113.2 (3)	O1—C16—C15	109.0 (4)
C6—N3—P2	124.2 (2)	O1—C16—H37	109.9
C5—N3—P2	122.4 (2)	C15—C16—H37	109.9
C7—N4—C8	112.3 (3)	O1—C16—H38	109.9
C7—N4—P3	119.2 (2)	C15—C16—H38	109.9
C8—N4—P3	124.7 (2)	H37—C16—H38	108.3
			
C22—C17—C18—C19	−1.5 (4)	C40—C35—B1—C23	154.8 (2)
B1—C17—C18—C19	177.8 (3)	C28—C23—B1—C17	148.3 (3)
C17—C18—C19—C20	1.0 (5)	C24—C23—B1—C17	−35.6 (3)
C18—C19—C20—C21	−0.2 (5)	C28—C23—B1—C29	27.4 (4)
C19—C20—C21—C22	0.0 (5)	C24—C23—B1—C29	−156.4 (3)
C20—C21—C22—C17	−0.6 (5)	C28—C23—B1—C35	−92.3 (3)
C18—C17—C22—C21	1.3 (4)	C24—C23—B1—C35	83.8 (3)
B1—C17—C22—C21	−178.1 (3)	N3—P2—N1—C2	148.8 (3)
C28—C23—C24—C25	1.9 (4)	N2—P2—N1—C2	33.0 (3)
B1—C23—C24—C25	−174.6 (3)	P1—P2—N1—C2	−90.5 (3)
C23—C24—C25—C26	−1.7 (5)	N3—P2—N1—C1	−60.6 (3)
C24—C25—C26—C27	0.4 (5)	N2—P2—N1—C1	−176.4 (2)
C25—C26—C27—C28	0.5 (5)	P1—P2—N1—C1	60.0 (2)
C26—C27—C28—C23	−0.2 (5)	N3—P2—N2—C3	170.1 (2)
C24—C23—C28—C27	−1.0 (4)	N1—P2—N2—C3	−79.5 (3)
B1—C23—C28—C27	175.4 (3)	P1—P2—N2—C3	38.3 (3)
C34—C29—C30—C31	−1.7 (4)	N3—P2—N2—C4	−22.2 (3)
B1—C29—C30—C31	178.7 (3)	N1—P2—N2—C4	88.3 (3)
C29—C30—C31—C32	1.6 (5)	P1—P2—N2—C4	−154.0 (2)
C30—C31—C32—C33	−0.1 (5)	N2—P2—N3—C6	−109.8 (3)
C31—C32—C33—C34	−1.2 (5)	N1—P2—N3—C6	135.2 (3)
C30—C29—C34—C33	0.2 (4)	P1—P2—N3—C6	21.8 (3)
B1—C29—C34—C33	179.8 (3)	N2—P2—N3—C5	75.9 (3)
C32—C33—C34—C29	1.2 (5)	N1—P2—N3—C5	−39.1 (3)
C40—C35—C36—C37	0.4 (4)	P1—P2—N3—C5	−152.5 (2)
B1—C35—C36—C37	−176.2 (3)	N6—P3—N4—C7	70.3 (3)
C35—C36—C37—C38	−0.3 (5)	N5—P3—N4—C7	−174.5 (2)
C36—C37—C38—C39	0.3 (4)	P1—P3—N4—C7	−42.0 (3)
C37—C38—C39—C40	−0.4 (4)	N6—P3—N4—C8	−85.9 (3)
C38—C39—C40—C35	0.5 (4)	N5—P3—N4—C8	29.3 (3)
C36—C35—C40—C39	−0.5 (4)	P1—P3—N4—C8	161.8 (2)
B1—C35—C40—C39	176.1 (3)	N6—P3—N5—C10	168.4 (2)
C18—C17—B1—C29	−104.7 (3)	N4—P3—N5—C10	52.7 (3)
C22—C17—B1—C29	74.7 (3)	P1—P3—N5—C10	−73.3 (3)
C18—C17—B1—C35	14.4 (4)	N6—P3—N5—C9	−21.8 (3)
C22—C17—B1—C35	−166.2 (3)	N4—P3—N5—C9	−137.5 (2)
C18—C17—B1—C23	134.1 (3)	P1—P3—N5—C9	96.5 (2)
C22—C17—B1—C23	−46.5 (3)	N4—P3—N6—C12	−160.7 (2)
C34—C29—B1—C17	−11.2 (4)	N5—P3—N6—C12	87.5 (3)
C30—C29—B1—C17	168.4 (2)	P1—P3—N6—C12	−42.6 (3)
C34—C29—B1—C35	−130.4 (3)	N4—P3—N6—C11	40.3 (3)
C30—C29—B1—C35	49.2 (3)	N5—P3—N6—C11	−71.6 (2)
C34—C29—B1—C23	109.6 (3)	P1—P3—N6—C11	158.4 (2)
C30—C29—B1—C23	−70.9 (3)	C16—O1—C13—C14	26.1 (5)
C36—C35—B1—C17	90.9 (3)	O1—C13—C14—C15	−26.5 (5)
C40—C35—B1—C17	−85.4 (3)	C13—C14—C15—C16	17.1 (6)
C36—C35—B1—C29	−149.5 (3)	C13—O1—C16—C15	−14.8 (6)
C40—C35—B1—C29	34.2 (3)	C14—C15—C16—O1	−1.7 (6)
C36—C35—B1—C23	−28.8 (3)		

### Hydrogen-bond geometry (Å, º)

**Table:**

*D*—H···*A*	*D*—H	H···*A*	*D*···*A*	*D*—H···*A*
C11—H33···O1^i^	0.98	2.52	3.454 (4)	158

Symmetry code: (i) −*x*+1, −*y*+1, −*z*.
